# Self-Handicapping in Chinese Medical Students During the COVID-19 Pandemic: The Role of Academic Anxiety, Procrastination and Hardiness

**DOI:** 10.3389/fpsyg.2021.741821

**Published:** 2021-09-17

**Authors:** Jun Jia, Lin-lin Wang, Jia-bin Xu, Xian-hao Lin, Bin Zhang, Qin Jiang

**Affiliations:** ^1^The School of Health, Fujian Medical University, Fuzhou, China; ^2^The School of Humanities and Management, Hunan University of Chinese Medicine, Changsha, China

**Keywords:** self-handicapping, academic anxiety, hardiness, procrastination, medical students

## Abstract

**Background:** In the face of the 2019 Coronavirus Disease (COVID-19) outbreak, Chinese medical students worried about their future studies which might make them more susceptible to academic anxiety. Previous studies have shown that academic anxiety is an important risk factor for self-handicapping, but there are few studies to explore the relationship between the two which may be mediated or moderated by other variables. Therefore, this study investigated how Chinese medical students' academic anxiety is correlated to their self-handicapping in time of COVID-19 epidemic, and explored the moderating and mediating effects of hardiness and procrastination.

**Methods:** In this study, 320 Chinese medical students' psychological traits were measured with Academic Anxiety Questionnaire, Self-Handicapping Scale, General Procrastination Scale and Hardiness Scale to explore the potential associations between these variables.

**Results:** The most obvious finding to emerge from this study was that self- handicapping had a positive correlation with academic anxiety and procrastination, but had a negative correlation with hardiness; hardiness had a negative association with academic anxiety and procrastination; and academic anxiety and procrastination were positively correlated. In addition, the relationship between academic anxiety and self-handicapping of Chinese medical students was not only partially mediated by procrastination, but also moderated by hardiness. Furthermore, medical students who had lower hardiness had stronger direct effect, while the indirect effect was strong at high and low conditions of hardiness.

**Conclusion:** In time of the COVID-19 epidemic, the academic anxiety and self-handicapping of medical students are influenced by procrastination and hardiness to a great extent. Thus, in addition to suggesting that more attention should be paid to the academic anxiety and procrastination of medical students, in the future, more attention should be paid to cultivating the hardiness of medical students and exerting its interventional role in self-handicapping.

## Introduction

On January 30th, 2020, the World Health Organization (WHO) announced a notice that the COVID-19 epidemic had been accounted for a public health emergency which aroused international concern (World Health Organization, 2019). Its characteristics such as highly infectious and rapid transmission had posed a threat to people's health, both physically and mentally. Faced with the infection which had become more and more serious, there were some countermeasures adopted by the Chinese government in order to control population flow and reduce infection, for instance, home quarantine and postponing the start of school year (Wang H. et al., 2020). However, due to the sudden long-term isolation, the reduction of social interaction and the change of learning style, college students might experience maladjustment, anxiety and other negative emotions. Students often experienced academic anxiety in learning situations (Collie et al., 2017; Lee, 2020). Some studies had shown that, compared to the ordinary college students, the medical students had a higher level of anxiety and depression under the impact of the epidemic of COVID-19 (Wang H. et al., 2020; Wang K. et al., 2020). At the same time, the level of anxiety in daily life and work continued to rise as the pandemic spread (Rodriguez et al., 2020). With the longer time of social isolation, people's anxiety and depression symptoms would become more prominent (Knopf, 2020). Indeed, academic anxiety had been proved to be significantly correlated with academic engagement and disengagement (Martin, 2008; Martin et al., 2012). Therefore, making a thorough inquiry of the academic anxiety level of medical students in time of COVID-19 epidemic and its psychological mechanism could provide evidence for future intervention.

Self-handicapping was the process of creating barriers to achieving successful performance designed to safeguard individuals' sense of self- competence (Jones and Berglas, 1978). The phenomenon that self-handicapping had a positive correlation with test anxiety was discovered in a qualitative study, and it was more likely that people with high self- handicapping were more likely to seek pretext for their procrastination, abandonment or failure (Martin et al., 2003). In other words, people with high self-handicapping had higher pressure before the exam, but they would not work very hard, so the final score was poorer than others (McCrea and Hirt, 2009). As mentioned above, people with high self-handicapping might procrastinate, deliberately lose learning materials, strategically give up efforts or avoid practicing skills in advance, which led to a decline in their academic performance (Martin, 2011; Putwain, 2019). As a major barrier to realizing personal potential and achievement, self-handicapping had many negative connections with physical and mental health and academic life (Barutçu and Demir, 2020). In time of the COVID-19 outbreak, applying the strategies of self-handicapping was one of the important factors affecting the physical and mental health development of medical students, which might lead to negative effects, such as poor learning status and lack of effort (Zarshenas et al., 2019). Therefore, for the purpose of enabling students to have a higher level of mental health and better academic performance during the COVID-19 epidemic, exploring their self-handicapping behaviors was worthwhile.

The emergence of the COVID-19 epidemic might aggravate symptoms such as academic anxiety and self-handicapping. Previous studies have explored the direct relationship between the academic anxiety and self-handicapping (Martin et al., 2003; McCrea and Hirt, 2009). However, it was still unclear about the potential mediating mechanism or moderating mechanism of academic anxiety affecting self-handicapping. In order to fill in these gaps, this study constructed a four-variable theoretical model to test whether and how the self-handicapping of Chinese medical students was affected by academic anxiety, procrastination, and hardiness. The way and time academic anxiety could affect self-handicapping would be advanced by the findings, and how to get rid of the negative influence of academic anxiety in time of the COVID-19 epidemic would also be known.

### The Mediating Role of Procrastination

Procrastination could be defined as the voluntary delay behavior of students in academic tasks though it was expected the consequences might be negative (Steel, 2007). Procrastination could be commonly found in the learning process of college students (Kim and Seo, 2015). Schraw et al.'s (2007) theoretical model showed that the fear of failure was one of the main reasons for academic procrastination. Furthermore, a number of studies had shown that procrastination was significantly related to anxiety, depression, life satisfaction, and self-efficacy (Balkis and Duru, 2016; Akpur, 2017; Ziegler and Opdenakker, 2018; Shi et al., 2019). Previous studies (Van Eerde, 2003; Wang, 2020) showed that test anxiety and procrastination were positively associated. When the students had a higher level of test anxiety, they also had a higher level of procrastination. Furthermore, Yerdelen et al. (2016) had investigated whether the students' anxiety and procrastination had a longitudinal association. Their survey suggested that procrastination substantially increased while academic anxiety decreased throughout the semester. When an academic term began, the procrastination of students and their anxiety had a positive association. It was likely that the students would manage their academic anxiety by procrastination in the short run, but this kind of procrastination would decrease when the final exams and deadlines came nearer because students wanted to avoid failure caused by inadequate preparation (Krispenz et al., 2019).

It had been a long time that procrastination was considered as a self- handicapping and dysfunctional behavior (Chu and Choi, 2005). Van Eerde (2003) had ever made a meta-analysis to examine the correlative factors of procrastination and his analysis found that procrastination and self- handicapping were closely related. Strunk and Steele (2011) explored the relative effects of different variables on procrastination behavior of college students, and they found that self-handicapping independently predicts procrastination. Furthermore, under laboratory conditions, Meyer also found that academic procrastination was a manifestation of self-handicapping, and there was a significantly positive correlation between them; thus, procrastination was one of the predictors of self-handicapping (Akin, 2012). When Beck et al. (2000) explored the effects of self-awareness and self-handicapping on academic procrastination, they found that there was a close relationship between academic procrastination and self-handicapping. Students with high self-handicapping and procrastination studied less, took longer to prepare for exams, and scored lower in the test. Therefore, self-handicapping and procrastination had similar structures, which could predict the score of each other. In addition, compared with procrastination, the concept of self-handicapping was more complicated and wider (Barutçu and Demir, 2020). All in all, procrastination could be predicted by academic anxiety, and procrastination and self-handicapping were significantly correlated with each other. Thus, the following hypothesis was proposed:

*Hypothesis 1:* During COVID-19, the level of self-handicapping of medical students will be affected by academic anxiety, and procrastination acts as a mediator between academic anxiety and self-handicapping.

### The Moderating Role of Hardiness

While academic anxiety might increase the level of procrastination and self-handicapping, not all medical students would be affected by it. Thus, it was critical to look for potential moderating factors, which might alleviate the relationship between academic anxiety and adverse effects. The purpose of this research was to investigate whether there were significant differences in self-handicapping or procrastination among medical students with different levels of hardiness.

Kobasa (1979) originally proposed hardiness as a hypothetical construct. Hardiness was regarded as a stable personality resource, which consisted of three cognitive factors, including commitment, challenge, and control. Commitment meant the capability of participating in life activities deeply, and it was the opposite of alienation. Control meant that an individual could influence the course of an event, and it was the opposite of powerlessness. Challenge epitomized people's normal expectations of life changes and promotion of further development, and it was the opposite of threats (Kobasa, 1979; Waysman et al., 2001). Furthermore, hardiness referred to an attribute of mental resilience, which had a relation to well-being, both physically and mentally and was ponderable in improving performance, meaningful behavior, and well-being under stress (Crowley et al., 2003; Maddi, 2014). Hardiness could mitigate the impact of stressors on stress, while hardiness and its components could improve the rating of school and job performance (Eschleman et al., 2010). This was because hardiness might make them less stressful by changing the perceptions of events and promote optimism and positive coping (Chan, 2000). Waysman et al.'s study (2001) found that hardiness had a moderating influence on the positive and negative changes in the long run after the traumatic things, and in times of stress, hardiness could help people to get practical help from others.

When dealing with epidemics, students might have different behaviors because of different levels of hardiness. Those students whose hardiness was higher responded to stresses through problem solving, rather than procrastinating to deny or avoid the present situation (Maddi et al., 2006). Thus, hardiness could be regarded as a positive personality characteristic which might make the academic anxiety less harmful. The effects of academic anxiety and procrastination on self-handicapping might be moderated by hardiness at the same time. Furthermore, according to previous studies on moderated mediation models (Edwards and Lambert, 2007; Hayes, 2018), when the direct influence of academic anxiety on self-handicapping was moderated by hardiness, and the mediating effect of procrastination was also moderated by hardiness, then it could be said that hardiness played a moderating role in the entire mediating model of procrastination. Following this, on the basis of the hypothesis that the influence of academic anxiety on self-handicapping might be mediated by procrastination, this paper further hypothesizes that hardiness might play a moderating role in it. Specifically, there were two hypotheses as follows:

*Hypothesis 2:* The direct influence of academic anxiety on self- handicapping is moderated by hardiness.
*Hypothesis 3:* The mediating role played by procrastination in academic anxiety and self-handicapping is moderated by hardiness.

### Current Study

**Figure 1:**
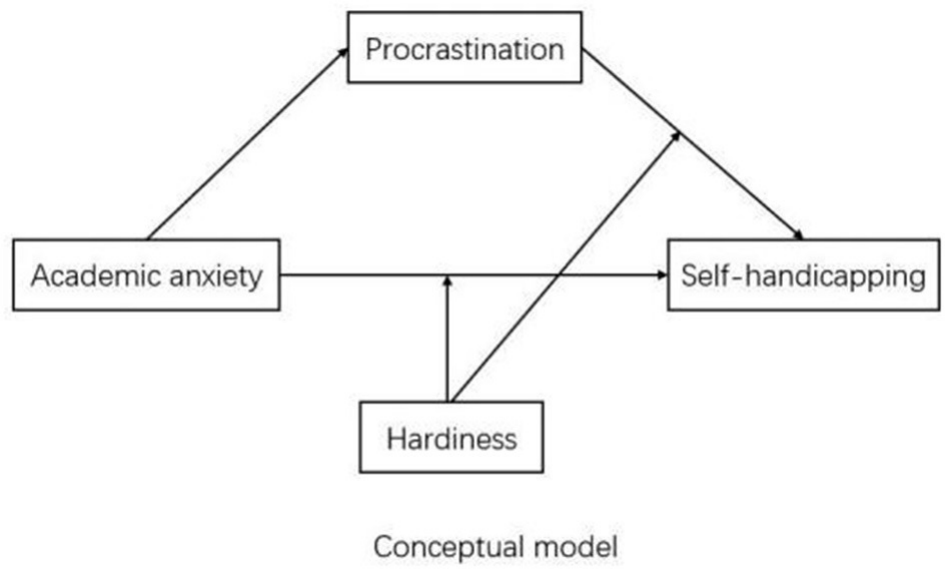
Conceptual model.

The paper explored the potential mechanism of the relationship between academic anxiety and self-handicapping of medical students. To be more specific, we answered three questions by examining a moderated mediation model: (a) Whether procrastination is a potential mediating variable of the effect of academic anxiety on self-handicapping, (b) Whether hardiness is a potential moderating variable of the effect of academic anxiety on self-handicapping, and (c) Whether the indirect mediating effect of procrastination on self-handicapping is moderated by hardiness. Compared with assessing two separate models, examining potential mediator and moderator variables in a single model at the same time could obtain more comprehensive information (Fairchild and MacKinnon, 2009). This study's moderated mediation model (see Figure 1) will show how and when academic anxiety affects self-handicapping, and how procrastination impacts self-handicapping. We hypothesized that self-handicapping is positively correlated with academic anxiety and procrastination, while negatively correlated with hardiness. The relationship between academic anxiety and self-handicapping is not only partially mediated by procrastination but also moderated by hardiness.

## Methods

### Participants

Three hundred and fifty students of Fujian Medical University in China participated in the study. Two polygraph questions were included in the questionnaire. The statements included “The following item was used to test whether you have answered the questions in a regular manner and please choose “4” directly out of 6 numbers ranging from 1 to 6 and “The following item is used to test whether you have answered the questions in a regular manner and please choose ‘5’ directly out of 6 numbers ranging from 1 to 6.” Thus it was guaranteed that the answers were authentic from the participants. Thirty students were excluded from data analysis because they failed to pass these two polygraph questions. Finally, 320 valid questionnaires were selected for the analysis of this research. Participants had a mean age of 20.83(SD = 0.70), ranging from 19 to 23. Of the participants, 33.4% were males, 66.6% were females; 6.9% were first grade, 12.8% were second grade, 71.6% were third grade, 7.8% were fourth grade, and 0.9% were students of fifth grades.

### Procedure

In order to avoid contact in time of the COVID-19 epidemic, our survey was made online on a Chinese survey website (www.wjx.cn). The questionnaires were completed in a voluntary and anonymous way. We randomly contacted the students of Fujian Medical University, and they agreed to help with our study. They were informed that they could withdraw from the study at any time. After obtaining the informed consent of participants, the participants completed the survey. The Biomedical Research Ethics Committee of Fujian Medical University approved our research content and data collection procedures (No. 79).

### Measures

#### Academic Anxiety Questionnaire

We used two items to measure medical students' levels of academic anxiety during COVID-19 on a 7-point scale ranging from 1 = “strongly disagree” to 7 = “strongly agree” (Oh et al., 2020). The statements included “I'm always nervous approaching the final exam” and “I'm anxious about the final exam.” Averaging the scores on the two items would calculate the total academic anxiety score, a high score indicated a high level of academic anxiety. Cronbach's α coefficient was 0.90 in this study.

#### Self-Handicapping Scale (SHS)

The Chinese version (Li and Yuan, 2004) of the Self-Handicapping Scale (SHS, Rhodewalt, 1990) was used to measure medical students' self-handicapping. This measure was a single- factor measure, which included 14 items (e.g., “When I do something wrong, my first impulse is to blame the circumstance”). The scale uses Likert's six-point score. Participants choose a number from 0 to 5, where 0 means strongly disagree and 5 means strongly agree. Averaging the score on the 14 items would calculate the total self-handicapping score, a high score indicated a high level of self-handicapping. Cronbach's α coefficient was 0.67 in this study.

#### General Procrastination Scale (GPS)

The Chinese version (Chu et al., 2010) of the General Procrastination Scale (GPS, Lay, 1986) was used to measure medical students' procrastination. This measure was a single- factor measure, which included 20 items (e.g., When preparing for deadlines, I often waste time doing other things). Each item was answered on a 5-point scale, ranging from 1 (do not apply to me at all) to 5 (apply to me very much). Averaging the score on the 18 items would calculate the total GP score, a high score indicated a high level of procrastination. Cronbach's α coefficient was 0.82 in this t study.

#### Hardiness Scale

The Chinese version (Lu and Liang, 2008) of the Hardiness Scale was used to assessed hardiness (Maddi et al., 2006). This measure included 27 items to assess four aspects of hardiness, including commitment, control, challenge and hardiness. Sample items included “When someone gets mad at me, I try to calm him down.” Participants responded on a four-point scale, ranging from 1 (do not apply to me at all) to 4 (apply to me very much). Mean scores were calculated, a high score indicated a high level of hardiness. Cronbach's α coefficient was 0.94 in this study.

### Data Analysis

The method of data collection was questionnaire survey. Thus, common method biases might show up. Therefore, we used Harman's single-factor testing to examine common method biases. The analysis suggested that there existed 16 factors and the eigenvalues was >1. The cumulative variance explained by the first factor was 20.69%, less than the critical value of 40%. From this, we could conclude that the study discussed in this paper had no serious common method biases.

The gender and grade difference analyses were first conducted in the present sample. SPSS 24.0 was used to calculate descriptive statistics and correlations of the study variables. We tested the moderated mediation model by Model 15 of the SPSS macro PROCESS version 3.0 (www.afhayes.com) developed by Hayes (2018). Many scholars used Model 15 to test moderated mediation models (e.g., Bartone and Homish, 2020; Jiang et al., 2021).

## Results

### Preliminary Analyses

It was suggested by the independent sample *t*-test that there was no grade difference between procrastination and self-handicapping, but significant grade differences could be seen in academic anxiety (*t* = −4.82, *p* < 0.001) and hardiness (*t* = 4.124, *p* < 0.001). To be more specific, males had a lower score in academic anxiety and a higher score in hardiness than females. One-way ANOVA showed that there was no significant difference in the levels of academic anxiety, procrastination and self-handicapping among students of different grades, but there was significant grade difference in hardiness (*F* = 2.42, *p* < 0.05). To be more specific, we found no significant differences in hardiness between freshmen and juniors, but seniors showed significantly higher levels of hardiness than the other grades. The mean, standard deviation and correlation coefficient of each variable were shown in Table 1. Academic anxiety had a positive correlation with procrastination and self-handicapping, and had a negative correlation with hardiness. Procrastination had a positive correlation with self- handicapping and had a negative correlation with hardiness. Hardiness had a negative correlation with self-handicapping.

**Table 1:** Descriptive statistics and inter-correlations between variables.

	**M**	**SD**	**1**	**2**	**3**	**4**	**Cronbach's α**
1. Academic anxiety	4.56	1.36	–				0.90
2. Procrastination	2.84	0.48	0.161^**^	–			0.82
3. Hardiness	2.44	0.51	−0.195^**^	−0.356^**^	–		0.94
4. Self-handicapping	3.31	0.49	0.242^**^	0.428^**^	−0.133^*^	–	0.67

*N = 320*.

* *p < 0.05*,

** *p < 0.01*.

### Testing for the Moderated Mediation Models

**Figure 2:**
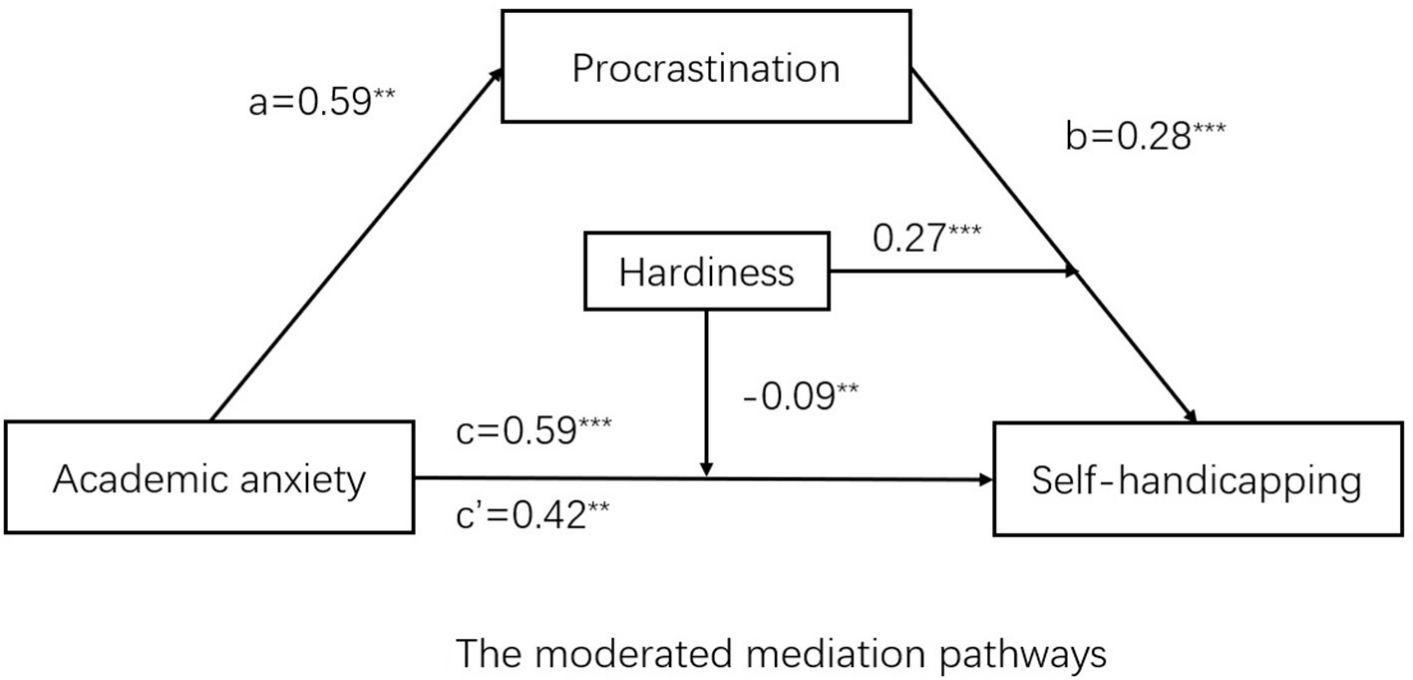
The moderated mediation pathways. **p < 0.01, ***p < 0.001.

**Figure 3:**
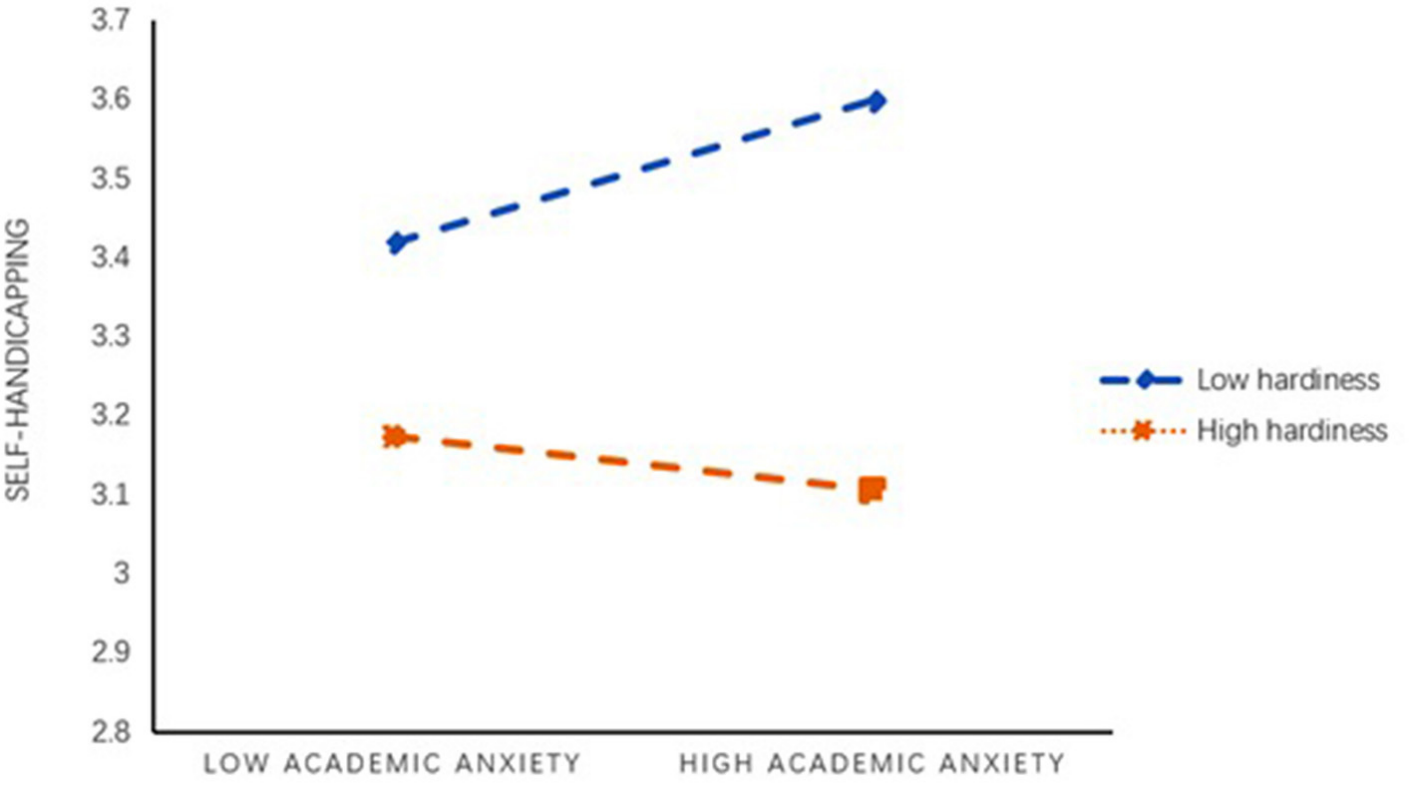
Hardiness moderates the relationship between academic anxiety and self-handicapping.

**Figure 4:**
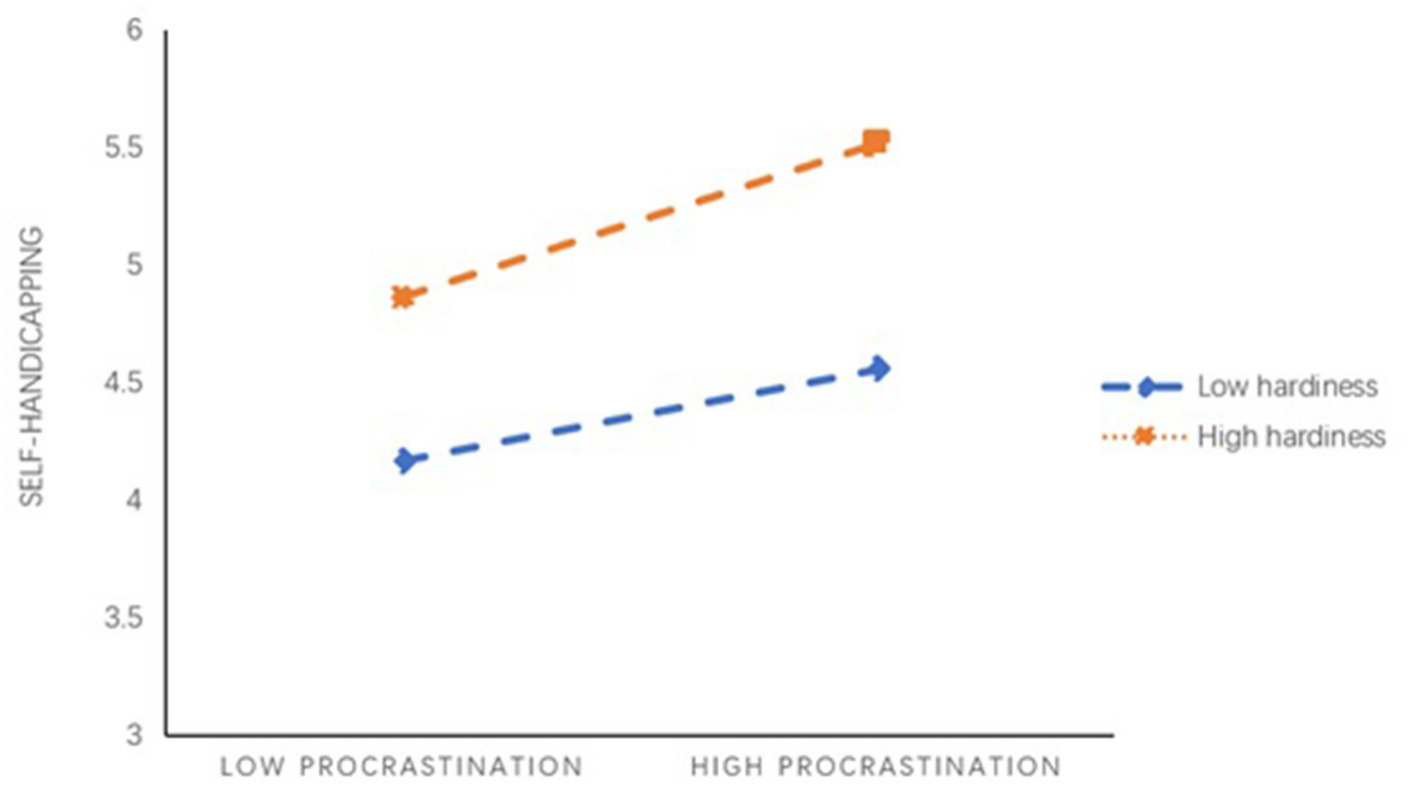
Hardiness moderates the relationship between procrastination and self-handicapping.

Controlling of gender and grade, model 4 in SPSS macro process version 3.0 compiled by Hayes was used to test the mediating effect of medical students' procrastination behavior between academic anxiety and self-handicapping. The results (see Table 2) showed that academic anxiety had a predictive effect on self-handicapping (*B* = 0.59, *t* = 4.11, *p* < 0.001), and the direct predictive effect of academic anxiety on self-handicapping was still significant (*B* = 0.42, *t* = 3.19, *p* < 0.01). Academic anxiety had a positive and predictive effect on procrastination (*B* = 0.59, *t* = 2.84, *p* < 0.05), and procrastination had a positive and predictive effect on self-handicapping (*B* = 0.28, *t* = 7.93, *p* < 0.001). In addition, the upper and lower limits of the bootstrap 95% confidence interval of the direct effect of academic anxiety on self-handicapping and the mediating effect of procrastination did not contain 0 (see Table 3), indicating that academic anxiety could directly predict the self-handicapping status of medical students, and indirectly predict the self-handicapping status through the mediating effect of procrastination. The direct effect (0.42) and intermediate effect (0.17) of the model accounted for 71.78 and 28.22% of the total effect (0.59). These results indicated procrastination partially mediated the relationship between academic anxiety and self-handicapping. Thus, H1 was supported. Controlling of gender and grade, model 15 in SPSS macro process version 3.0 compiled by Hayes was used to examine the moderating effect of hardiness. The results suggested that the interaction of academic anxiety and hardiness showed significant effects on self-handicapping (*B* = −0.09, *p* < 0.01), and the interaction of procrastination and hardiness showed significant effects on self-handicapping (*B* = 0.27, *p* < 0.001) (see Table 4). These findings suggested that the effects of both academic anxiety and procrastination on self-handicapping were moderated by hardiness (see Figures 2–4). Moreover, as could be seen from Table 5, two of the three conditional direct effects (based on the moderator values at the mean and at −1 standard deviation) and three conditional indirect effects had a positive and significant difference from zero. Namely, the effect of academic anxiety on self-handicapping was observed when hardiness was moderated to low, but not when hardiness was high. The indirect effect of academic anxiety on self-handicapping through procrastination was observed when hardiness was moderated to low and high. Thus, both H2 and H3 were supported.

**Table 2:** The mediation model of procrastination.

**Outcome variable**	**Predictor variable**	***R^**2**^***	**F**	***B***	***t***	**LLCI**	**ULCI**
Self-handicapping		0.06	6.86^***^				
	Sex			0.53	0.65	−1.09	2.16
	Grade			0.34	0.64	−0.71	1.39
	Academic anxiety			0.59	4.11^***^	0.31	0.87
Procrastination		0.03	2.87^**^				
	Sex			−0.29	−0.25	−2.63	2.04
	Grade			−0.27	−0.35	−1.79	1.24
	Academic anxiety			0.59	2.84^**^	0.18	0.99
Self-handicapping		0.22	21.89^***^				
	Sex			0.62	0.82	−0.87	2.10
	Grade			0.42	0.86	−0.54	1.38
	Academic anxiety			0.42	3.19^**^	0.16	0.68
	Procrastination			0.28	7.93^***^	0.21	0.35

*N = 320*.

** *p < 0.01*,

*** *p < 0.001. LL, Low limit; CI, 95% confidence interval; UL, upper limit*.

**Table 3:** Path coefficients for mediation model.

**Path coefficients**			**Effects**		
	**Self-handicapping (Y)**	**Procrastination (M)**		**95% CI**	
				**Lower**	**Upper**
Academic anxiety	c' = 0.42^**^	a = 0.59^**^			
Procrastination	b = 0.28^***^				
Specific: Academic anxiety → Self-handicapping			c' = 0.42	0.10	0.75
Specific: Academic anxiety → Procrastination → Self-handicapping			ab = 0.17	0.04	0.31

*N = 320. Bootstrap sample size = 5,000. 95% CI, 95% confidence interval*.

** *p < 0.01*,

*** *p < 0.001*.

**Table 4:** The moderation model of hardiness.

**Outcome variable**	**Predictor variable**	***R^**2**^***	**F**	***B***	***t***	**LLCI**	**ULCI**
Self-handicapping		0.26	15.82^***^				
	Sex			0.04	0.73	−0.93	2.04
	Grade			0.02	0.49	−0.71	1.18
	Academic anxiety			0.07	3.55^***^	0.21	0.72
	Procrastination			0.41	7.61^***^	0.21	0.36
	Hardiness			0.05	0.91	−0.03	0.08
	Academic anxiety × hardiness			−0.09	−3.19^**^	−0.04	−0.01
	Procrastination × hardiness			0.27	3.48^***^	0	0.01

*N = 320*.

** *p < 0.01*,

*** *p < 0.001. LL, Low limit; CI, 95% confidence interval; UL, upper limit*.

**Table 5:** Direct effect and mediating effect on different levels of hardiness.

	**Hardiness**	**β**	**Boot SE**	**Boot LLCI**	**Boot ULCI**
Direct effect	M−1 SD (1.93)	0.11	0.02	0.06	0.16
	M (2.44)	0.07	0.02	0.03	0.1
	M+1 SD (2.95)	0.02	0.02	−0.03	0.06
Mediation effect	M−1 SD (1.93)	0.02	0.01	0	0.03
	M (2.44)	0.02	0.01	0.01	0.04
	M+1 SD (2.95)	0.03	0.01	0.01	0.06

*N = 320. Bootstrap sample size = 5,000. LL, Low limit; CI, 95% confidence interval; UL, upper limit*.

## Discussion

This paper analyzed the mechanisms underlying the association between academic anxiety and self-handicapping by constructing a moderated mediation model among medical students in time of the COVID-19 epidemic. This study found that, in the relationship between academic anxiety and self-handicapping, procrastination played a mediating role, and that hardiness played a moderating role. Furthermore, hardiness also moderated the mediating effect of procrastination. Taking into account the negative effects of academic anxiety and self-handicapping, more attention should be paid to the psychological conditions of medical students in time of the COVID-19 epidemic, so as to intervene in self-handicapping by cultivating hardiness in an appropriate way.

Firstly, our study found that self-handicapping would be significantly predicted by academic anxiety among medical students. Our research result was consistent with the result of previous studies which reported that academic anxiety had a positive association with self-handicapping (Thomas and Gadbois, 2007). In addition, because students were affected by isolation in time of the COVID-19 epidemic, they could only learn via the Internet to replace ordinary face-to-face courses in the classroom. Therefore, teachers could not give immediate feedback on students' learning. Due to the changes in the study, students underwent academic anxiety as the result of their fear toward failure. The self-worth theory (Covington, 1992) suggested that students' self-worth could be affected by failure, because failure was considered a symbol of low ability, which was considered the same as low self-worth (Martin et al., 2001). In the present educational environment, the emphasis on individual achievement and performance, as well as perceptions of self-worth, was heightened (Leondari and Gonida, 2007; Gadbois and Sturgeon, 2011). Consequently, students whose level of academic anxiety was higher were willing to adopt self-handicapping and strategies of defensive pessimistic, which could protect their sense of self-worth from expected failure by reflecting the attribution away from their perceived incompetence (Covington, 2009; Putwain, 2019). Although some of the previous studies have indicated that positive outcomes such as improving performance and enjoyment, minimizing the impact of failure, and preparing people for others' evaluations in case that they might fail would be provided by self-handicapping (Rhodewalt and Hill, 1995; Deppe and Harackiewicz, 1996; Brown and Kimble, 2009), Martin et al. (2001) study showed that, compared with defensive and pessimistic strategies, self-handicapping had a higher degree of negative correlation with educational outcomes (Thomas and Gadbois, 2007). Therefore, high learning anxiety not only led to students' self-handicapping, but also caused students' poor academic performance.

Secondly, the results confirmed that the relationship between academic anxiety and self-handicapping would be partially mediated by procrastination. Studies in the past have revealed that there was a significant connection between procrastination, academic anxiety and self-handicapping (Akin, 2012; Yerdelen et al., 2016), but as far as we know, there was no research which had proved the indirect relationship between academic anxiety and self-handicapping through the mediating effect of procrastination. Our research suggested that academic anxiety had an influence on procrastination, and procrastination mediated the impact of academic anxiety on self-handicapping to a certain extent, and this finding was in consistency with our previous hypothesis. In addition, long-term online learning would increase the time students spend on the Internet, and a high degree of Internet dependence might have negative effects on college students, such as causing anxiety (Lai et al., 2015), social isolation (Shaw and Black, 2008), a decrease in self-confidence (Steel, 2007), and even Internet addiction (Hayat et al., 2020). Research in the past had recognized barriers to online learning environments, such as issues of self-regulation, self-pacing, learning speed, and differences in effort (Azevedo et al., 2005; Hooshyar et al., 2019). Therefore, in time of the quarantine, students were still unable to compare their own learning progress with others and their fear and anxiety increased as a result, which would cause academic procrastination.

Furthermore, the results of this investigation showed that hardiness could moderate both the effect that academic anxiety itself exerted on self- handicapping and the effect that academic anxiety exerted on self- handicapping through procrastination. The direct effect of medical students who had low hardness was stronger, and the indirect effect of medical students with both high and low hardness was stronger. That is, hardiness not only reduced the direct influence of academic anxiety on self- handicapping, but also remitted the indirect influence of academic anxiety on self-handicapping via procrastination. These findings were consistent with research in the past revealing the protective effects of academic anxiety and self-efficacy on hardiness (Abdollahi and Noltemeyer, 2018; Abdollahi et al., 2018; Cheng et al., 2019).

To be more specific, the academic hardiness theory (Benishek et al., 2005) held that medical students who had high levels of academic hardiness had less procrastination. They believed it was a chance for growth and development, rather than a stressful event for students to learn online in time of the COVID-19 epidemic. Furthermore, they were confident and able to continue to study hard and pursue academic goals (Abdollahi et al., 2018). There were an internal locus of control, effective learning strategies, time management skills, achievement orientation, and low levels of academic stress within the students who had high academic hardiness (Abdollahi et al., 2020a,b). This kind of students were unlikely to avoid academic tasks by adopting procrastination or self-handicapping strategies. In time of the COVID-19 epidemic, facing a harsh academic environment, students with high academic hardiness tended to assess this situation as more challenging, controllable and less threatening, and they were easier to adapt and participate in situations to learn more and thrive even under pressure (Kamtsios and Karagiannopoulou, 2015). Students who had high academic hardiness also used more emotional self-regulation skills, which could help them optimistically and actively participate in academic tasks, and challenged their arduous courses to gain experience under pressure (Abdollahi et al., 2015). Therefore, medical students with high hardiness could better deal with academic anxiety.

## Limitations and Implications

There were a few limitations in this study, which demanded to be improved in consecutive research. Firstly, the study used a cross-sectional study design; therefore, no causal explanation could be obtained. Consecutive research should use longitudinal design or experimental research to explore the causal relationship between academic anxiety and self-handicapping by using cross design, multi-layer linear model or by manipulating the independent variables and mediating variables. Secondly, we used the self-report questionnaires to explore the role of academic anxiety, procrastination, and hardiness. Consecutive research should conduct interventions for students and their families. Thirdly, the participants in this study came from Fujian Province of China. Fujian Province is a low-risk area for COVID-19. Moreover, this study did not collect data right before the final exam. Thus, the response of medical students might be affected by the time and place of data collection. Finally, this research only assessed procrastination as a mediating factor and hardiness as a moderating factor. Thus, consecutive studies are demanded to assess other potential factors to improve its robustness. Future research can aim to address these limitations.

Although this study had some limitations, it was the first study to investigate academic anxiety and self-handicapping of medical students in time of the COVID-19 epidemic, and it also explored the mediating role of procrastination and the moderating role of hardiness. The exploration of the underlying mechanism of this relationship would enrich the existing research results. In particular, this study explained how and when academic anxiety affected self-handicapping, and how procrastination impacted self-handicapping. In addition, there were some important practical implications. First, parents and educators must be aware of the academic anxiety of medical students in time of the COVID-19 epidemic and its negative effects in order to help them reduce their academic anxiety over time. Second, given that procrastination was a significant mechanism linking academic anxiety and self-handicapping, it should be effective for educators and psychologists to help medical students to develop good study habits to reduce the behavior of procrastination. And if we could combine psychoeducational, behavioral, and cognitive interventions, a positive effect could be achieved (Van Eerde and Klingsieck, 2018; Malouff and Schutte, 2019). For instance, some studies have suggested that the way to meddle in medical students' procrastination through the internet-based acceptance and commitment therapy and cognitive behavioral therapy which were effective and acceptable (Gagnon et al., 2019; Küchler et al., 2019). Besides, the need to develop specific support plans for medical students' procrastination behavior was emphasized, including improving academic skills, increasing self-regulated learning strategies, and reducing emotional state problems (Hen, 2018). Thirdly, the direct influence of academic anxiety on self-handicapping could be alleviated by hardiness, but the indirect influence of academic anxiety on self- handicapping through the mediating role of hardiness could also be alleviated by hardiness. Children's tough personality could be affected by parental education styles (Higinio and Antonia, 2020). Moreover, previous studies had suggested that the hardiness of nurses, as a positive personal resource, could reduce nurses' stress and burnout, influenced the way nurses interacted with the working environment, and improved nurses' input (Bemana et al., 2014; Garrosa et al., 2014). Thus, to improve medical students' hardiness might be a good method for buffering the negative impacts of academic anxiety.

## Conclusion

The association between academic anxiety and self-handicapping was partially mediated by the level of procrastination of medical students during the COVID-19 pandemic. In addition, hardiness not only moderated the effect of academic anxiety on self-handicapping of medical students, but also moderated the mediating effect of procrastination. Specifically, it moderated the effect of procrastination on self-handicapping. As academic anxiety and self-handicapping had a negative impact on medical students, it was necessary to pay close attention to their physical and mental conditions, and appropriate intervention could be taken with the self- handicapping of medical students by cultivating hardiness.

## Data Availability Statement

The original contributions presented in the study are included in the article/Supplementary Material, further inquiries can be directed to the corresponding author/s.

## Ethics Statement

The studies involving human participants were reviewed and approved by Biomedical Research Ethics Committee of Fujian Medical University (No. 79). The patients/participants provided their written informed consent to participate in this study.

## Author Contributions

The idea for this study was conceived by QJ, JJ, and BZ. QJ and JX collected data. JJ made an analysis and interpretation of the data. JJ and LW wrote the manuscript. QJ revised the manuscript. The final version was read and approved by all authors.

## Funding

This study was supported by School of Health of Fujian Medical University, Research topics of Ideological and Political Work of Fujian Medical University (70000049).

## Conflict of Interest

The authors declare that the research was conducted in the absence of any commercial or financial relationships that could be construed as a potential conflict of interest.

## Publisher's Note

All claims expressed in this article are solely those of the authors and do not necessarily represent those of their affiliated organizations, or those of the publisher, the editors and the reviewers. Any product that may be evaluated in this article, or claim that may be made by its manufacturer, is not guaranteed or endorsed by the publisher.
