# Supplementary material for: Self-Handicapping in Chinese Medical Students During the COVID-19 Pandemic: The Role of Academic Anxiety, Procrastination and Hardiness
Source: Front Psychol. 2021 Sep 17;12:741821. doi: 10.3389/fpsyg.2021.741821 (PMC8484870; doi:10.3389/fpsyg.2021.741821)
Supplement: Supplementary file 1 [file Data_Sheet_1.pdf]

Appendix:

Questionnaires

Academic anxiety questionnaire

1.I am always nervous approaching the final exam

(Level 1-7, 1=the least nervous, 7=the most nervous)

1 2 3 4 5 6 7

2.I am very anxious about the final exam

(Level 1-7, 1=the least anxious, 7=the most anxious)

1 2 3 4 5 6 7

The Self-Handicapping Scale

①Completely disagree ②Strongly disagree ③A little bit disagree
④A little bit agree ⑤Strongly agree ⑥Completely agree

1.Whenever I do something wrong, I always think it is caused by the bad environment

2.I'm used to putting things off until the end

3.I think I am more vulnerable to the environment than most people

4.No matter what it is, I will try my best to do it

5.I am easily distracted by noise or my creative ideas

6.I try not to put too much enthusiasm into competitive activities, so that if I fail or do badly, It won't hurt me too much

7.If I try harder, I will do better

8.Sometimes I want to have a minor illness for a day or two because it can reduce stress

9. I would have done better if I hadn't been in a bad mood

10.I admit that when I fail to live up to the expectations of others, I often find excuses and reasons why it's not my fault

11.I often think that I didn't get good results in sports because I was less lucky than others

12.I often overeat

13.I never let emotional problems interfere with other aspects of my life

14.Sometimes I feel so depressed that simple work becomes difficult

Polygraph Question

The following item is used to test whether you have answered the questions in a regular manner and please choose‘4’directly”

1 2 3 4 5 6

The General Procrastination Scale

①do not apply to me at all ②part of it doesn't apply to me ③not sure ④part of it does apply to me ⑤apply to me very much

1.I often do what I planned to do a few days ago

2.I finished my homework before I had to hand it in

3.After reading the borrowed books, I will return them to the library immediately whether they are due or not

4.When it's time to get up in the morning, I always get up right away

5. After I've written the letter, I'll probably sit it out for a few days

6.I always call back quickly

7.Even very simple and easy work, I seldom finish it in a few days

8.I usually make decisions quickly

9.I always put off what I have to do

10.I usually rush to finish my homework on time

11.When I go out, I always get ready in advance

12.When preparing for the deadline, I often waste time doing other things

13.I prefer to keep the appointment in advance

14.I usually start to do it soon after my homework is assigned

15.I usually finish the task ahead of time

16.I always seem to buy birthday or holiday gifts at the last minute

17.Even for necessities, I usually wait until the last minute to buy them

18.I usually finish everything planned in a day

19.I often say "do it tomorrow"

20.I usually deal with all the tasks that must be completed before entertainment and leisure in the evening

Polygraph Question

The following item is used to test whether you have answered the questions in a regular manner and please choose '5' directly.

1 2 3 4 5 6

The Chinese version of the Hardiness Scale

①do not apply to me at all ②part of it doesn't apply to me

③part of it does apply to me ④apply to me very much

1. Breaking the routine will inspire me to study
2. When someone is angry with me, I will try to calm him down
3. I always put great enthusiasm into my work
4. I am not afraid of any difficulty in deciding what to do
5. Work and study will bring me fun
6. Even if it is very simple, I will do it very hard
7. I can keep my spirits up even when things are not going well
8. The busy pace of life makes me feel a sense of fulfillment
9. Whenever there is a problem, I will try my best to find the root cause
10. In the face of disadvantage, I will try to turn the situation around
11. I will keep calm in the face of criticism from others
12. I'm really excited to be able to work hard
13. If the goal has been determined, I will not give up even if there are obstacles
14. The changes in life and work often cheer me up
15. I will not give up my ideals and pursuit easily
16. I often regard the difficulties encountered in life as a challenge rather than a threat
17. I prefer to take on important work
18. I look forward to working / studying almost every day
19. I'd rather do those challenging and changing jobs
20. I like to try new and exciting things
21. I am willing to give up a stable life in order to get the opportunity to face major challenges
22. I can always achieve my goals through my own efforts
23. No matter how complicated the problem is, I can always clear my mind quickly
24. When I encounter difficulties, I always try my best to find a solution
25. It's important to meet new situations in my life
26. With hard work, any difficulty can be overcome
27. As long as it is meaningful, no matter how difficult it is, I can stick to it
